# Shape-Tunable UV-Printed Solid Drugs for Personalized Medicine

**DOI:** 10.3390/polym14132714

**Published:** 2022-07-02

**Authors:** Bobby Aditya Darmawan, Sang Bong Lee, Minghui Nan, Van Du Nguyen, Jong-Oh Park, Eunpyo Choi

**Affiliations:** 1 School of Mechanical Engineering, Chonnam National University, 77 Yongbong-ro, Buk-gu, Gwangju 61186, Korea; bobbyaditdit@gmail.com (B.A.D.); myeonghea94@kimiro.re.kr (M.N.); nvdu81@gmail.com (V.D.N.); 2 Korea Institute of Medical Microrobotics, 43-26, Cheomdangwagi-ro 208-beon-gil, Buk-gu, Gwangju 61011, Korea; 3 THERABEST, Co., Ltd., Seocho-daero 40-gil, Seoul 06657, Korea; sblee@therabest.co.kr

**Keywords:** hydrogel, solid drugs, UV-patterned

## Abstract

Several recent advances have emerged in biotherapy and the development of personal drugs. However, studies exploring effective manufacturing methods of personal drugs remain limited. In this study, solid drugs based on poly(ethylene glycol)diacrylate (PEGDA) hydrogel and doxorubicin were fabricated, and their final geometry was varied through UV-light patterning. The results suggested that the final drug concentration was affected by the geometrical volume as well as the UV-light exposure time. The analysis of PEGDA showed no effect on the surrounding cells, indicating its high biocompatibility. However, with the addition of doxorubicin, it showed an excellent therapeutic effect, indicating that drugs inside the PEGDA structure could be successfully released. This approach enables personal drugs to be fabricated in a simple, fast, and uniform manner, with perfectly tuned geometry.

## 1. Introduction

Synthetic polymers are a diverse and promising class of biomaterials with a wide range of applications, including implants, wound dressings, drug delivery systems, or materials for tissue engineering [1,2]. These polymers tend to provide versatility in terms of functionality, softness, recoverable strain, and lighter weight [3]. Among polymeric materials, hydrogels have been used in many biomedical applications on account of their tunable mechanical and chemical properties, and biocompatibility [4]. Hydrogels are multi-dimensional polymer networks that are saturated with water. The quantity of water can be as high as 99 wt% of the hydrogel mass [5,6]. In particular, poly(ethylene glycol)diacrylate (PEGDA), a PEG-based polymeric hydrogel, is easily synthesizable and exhibits various benefits such as good biocompatibility, tissue-like properties, high-temperature and pH stability, and reactive sites that enable chemical modification and easy tunability. This makes it suitable for a variety of biomedical applications, such as drug delivery systems [7,8,9,10,11].

Photolithography has been used in microfabrication, which includes the transfer of geometric shapes from a film mask into a thin-layer surface through UV irradiation on a photo-sensitive polymer; this method has been proven as a powerful tool for patterning materials with high resolution, smooth surface finish, and avoidance of drug thermal degradation [12,13]. As a result of recent advances in biotherapy and personalized medicine, novel formula (e.g., nanoscale medicines, biomimetic particles, functionalized liposomes) as well as more sophisticated manufacturing methods have emerged [14]. Therefore, for personal medicines to be effective, they should be easily fabricated, customizable, and affordable [15]. According to the American Society for Testing and Materials, there are seven common techniques for printing in medicines, i.e., binder jetting, vat photopolymerization, stereolithography, digital light processing, material extrusion processes, fused deposition modelling, which utilizes thermoplastics, and semisolid extrusion based on the use of gels and pastes [16]. Several studies have been conducted on various personal medicine approaches; for instance, Clark et al. developed 3D-printed drugs based on PEGDA hydrogel [17]. Ayyoubi et al. reported the fabrication of spherical mini tablets loaded with nifedipine (NFD) through a 3D printing process [18]. Although there is much research and development in the fabrication of personalized drugs, design challenges and the complex 3D printer requirements to fabricate the drugs are still the major limitations of applications of 3D printing for personalized medicine.

**Figure 1:**
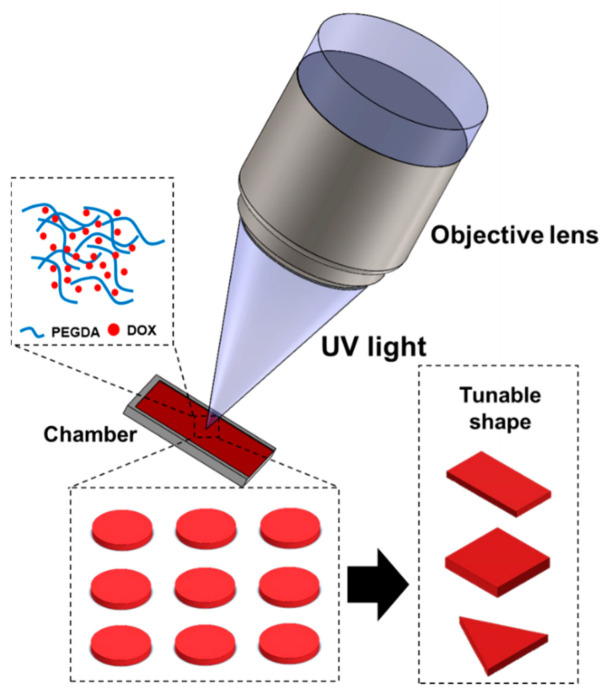
Schematic illustration of the proposed study: UV light is used to pattern the polymer.

Therefore, in this study, the solid drug was designed using PEGDA hydrogel through simple, easy, and relatively fast fabrication. As illustrated in Figure 1, PEGDA (MW 575) was selected as the main material and doxorubicin (DOX) as the drug for this study. DOX has been widely used as a chemotherapeutic drug to treat numerous types of cancer. Moreover, DOX is a very potent drug and a cost-effective compound compared to other therapeutic agents [19,20,21,22]. In particular, a photoinitiator is added into the prepolymers for photopolymerization [23], the photoinitiator is important in printing efficiency [24]. Therefore, the photoinitiator was mixed into the prepolymer solution. Through the application of UV light, the solid drugs were fabricated into various shapes. In addition, the effect of the geometrical volume on the final drug concentration was evaluated. Finally, the fabricated solid drugs were characterized, and the drug release profile was observed and evaluated as per in vitro cell studies. Thus, with its easy customizability and excellent biocompatibility, PEGDA can be a promising candidate to be used together with drugs to create personalized medicine.

## 2. Materials and Methods

### 2.1. Material Preparation

Polyethylene glycol diacrylate (PEGDA; MW 575) and lithium phenyl-2,4,6-trimethyl benzoyl phosphinate (LAP) photoinitiator were purchased from Sigma Aldrich (St. Louis, MO, USA), and doxorubicin was purchased from MedKoo Biosciences (Morrisville, NC, USA). For the material preparation, 1 mL of PEGDA (MW 575) and 10 mg of LAP photoinitiator were first added to 1 mL of DI water. The mixture was vortexed for 30 min. Then, 10 mg of doxorubicin was added and the solution was vortexed again for 10 min. The precursor was kept in low-light conditions to avoid light exposure.

### 2.2. Fabrication

The PEGDA was selected as a base material, and doxorubicin as the model drug. The solid drugs were fabricated using focused UV light with a fixed power of 12 µW and wavelength of 365 nm. In summary, the UV LED of the optical system illuminated the chamber through an objective lens. A film mask was placed between the condenser and doublet lenses to pattern the desired shape. The details of the system are listed in our previous works [25,26,27] and the digital photograph of the system is shown in Figure S1. Initially, the microchamber was fabricated by the 3D printer (25 mm × 25 mm) and the slide glass of the desired thickness (300 µm) was placed above the microchamber, as illustrated in Figure S2. Subsequently, the precursor material was injected into the microchamber through capillary force and directly placed in the workspace. Before the fabrication process, an objective lens was focused on the desired position, and the surface was clearly seen. UV light (λ = 365 nm) was used to pattern the drugs for various shapes and sizes. The UV irradiation process was performed for 30 s and the patterned solid was cleaned using DI water directly to remove unpolymerized materials.

### 2.3. Characterization

The scanning electron microscope (SU8010, Hitachi, Tokyo, Japan) used secondary electrons operated at the voltage of 2 keV. The energy dispersive spectroscopy (EDS) analysis was performed at a voltage of 15 keV. Fourier transform infrared spectrometry (FTIR; EMPYREAN, PANalytical Co., Malvern, UK) was used to evaluate the chemical bonds and specific functional groups. The samples were analyzed at a range of wavenumbers from 4000 cm^−1^ to 400 cm^−1^. In addition, the thermal properties of the samples were investigated through a thermogravimetric analyzer (TGA; DTG-60H, Shimadzu, Kyoto, Japan). The samples were placed in the alumina pan at a constant rate of 10 °C/min in a 50 mL/min flow of nitrogen; the temperature range was set from 25 °C to 700 °C. The TGA results were analyzed by using Origin software.

### 2.4. Swelling Test

The swelling test is an essential parameter in evaluating the hydrogel’s characteristics. The swelling is defined as the quantity of water or buffer absorbed by the hydrogel [28]. Here, the swelling ratio was evaluated by measuring the ratio of the mass of swollen samples (*M_S_*) to the dried samples (*M_D_*) for solid drugs in various UV exposure time. For the swollen samples, the solid drugs were swollen in PBS at room temperature for at least 24 h. Meanwhile, for the dried samples, the solid drugs were freeze-dried in a freeze dryer (FDCF-12003, Operon) for 24 h. The mass swelling ratio was calculated as:

(1) $$\mathrm{Mass}\;\mathrm{swelling}\mathrm{ratio}=\frac{M_{S}}{M_{D}}$$

### 2.5. Drug Concentration and Drug Loading Capacity Measurements

The drug present in each sample was measured immediately after fabrication by dissolving the patterned solid drugs in 1 mL of DI water by using a probe sonicator for 2 min in an ice bath to avoid evaporation. Then, 100 μL of the dissolved solution was taken, and the fluorometric value was measured through a microplate reader.

The drug loading capacity was measured by calculating the mass of the loaded drug (µg) to the mass of the solid drugs (mg), as reported by Eslami et al. [29]. The measurements were conducted for each UV exposure time.

### 2.6. Drug Release Experiments

The drug release of doxorubicin was evaluated in two different pH conditions: pH 5.5 and pH 7.4. Here, five solid drugs were added into 5 mL of solution. At the desired point in time, 100 μL of the released media was taken and the absorbance measured by using a microplate reader. The same amount of solution was refilled. The drug content was calculated based on a standard curve developed for doxorubicin. The cumulative release was calculated with the following equation:

(2) $$\mathrm{Cumulative}\;\mathrm{release}\;(\%)=\frac{D_{t}}{D_{0}}\;\times \;100$$

where *D_t_* represents the amount of drug released at the time *t*, and *D*_0_ represents the total amount of drug present in the sample.

### 2.7. In Vitro Cell Studies

To check the biocompatibility of the materials, doxorubicin-free PEGDA was patterned and prepared. A normal mouse (*Mus musculus*) lung cell (MLg; KCLB No. 10206), human derived stomach cancer cells (MKN45; KCLB No. 80103) and immortalized human hepatocyte cell (Fa2N-4; ATCC No. PTA-5566) were cultured, seeded in a 48-well plate, and incubated overnight (CO_2_ 5%; 37 °C). An increasing number (one, two, and five) of UV-patterned PEGDA samples were then treated with the cells. After 24 h, the cells were removed, treated with 100 μL of thiazolyl blue tetrazolium bromide (MTT, 0.5 mg/mL) in Dulbecco’s modified Eagle Medium, and further incubated for 4 h. Finally, the media were removed and replaced by 100 μL of dimethyl sulfoxide (DMSO). The cell viability was observed by using a microplate reader (λ = 570 nm).

To check the effectivity of the solid encapsulated drugs, those solid drugs were treated against cancer cells (stomach cancer cells [MKN45; KCLB No. 80103] and colon cancer cells [CT26; KCLB No. 80009]) through MTT assay. The MKN45 and CT26 cells were cultured and seeded in separate 48-well plates and incubated for 24 h (CO_2_ 5%; 37 °C). Subsequently, increasing numbers of solid drugs (two and five) were added and incubated for another 24 h. Finally, the cells were treated with MTT and then dissolved in DMSO, as was mentioned earlier. The cell viability was checked and evaluated with the microplate reader (λ = 570 nm). A live/dead cell assay was conducted to visualize the cells after treatment through a calcein-AM/ethidium homodimer-1 (EthD-1) co-staining kit (Thermo Fisher Scientific, Waltham, MA, USA), which was done based on the manufacturer’s protocols. Finally, the live/dead cells’ images were taken using a confocal microscope.

### 2.8. Statistical Analysis

At least three repeated quantitative measurements were taken in each sample group, and the statistical data were analyzed using Student’s *t*-test. Means and standard deviations were used to present quantitative values. It should be noted that the symbol ** indicates *p* < 0.01, and * indicates *p* < 0.05, implying statistically significant results.

## 3. Results and Discussion

### 3.1. Fabrication and Characterization

**Figure 2:**
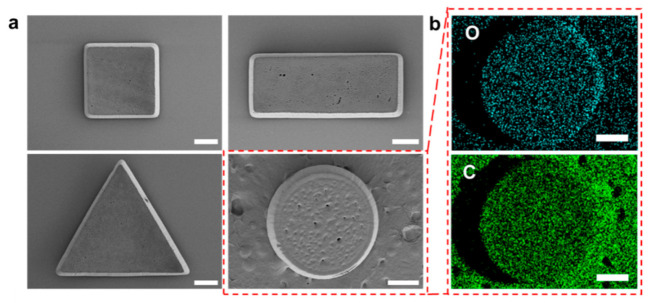
SEM–EDS images of the patterned PEGDA: (**a**) SEM images of the patterned PEGDA for various shapes, namely square (40×), triangle (40×), rectangular (30×), and circular (30×); (**b**) EDS images of circular-shaped solid drugs (scale bar: 500 μm).

The solid drugs were fabricated by using an optical system as mentioned in the previous section. Figure S3 shows the optical microscope images of both drug-free and the solid drug complexes, where the drug-free (PEGDA) UV-patterned solid has a white color, and the solid drug has a red color that comes from the doxorubicin. In addition, Figure 2a shows the SEM images of the solid drug that were fabricated in various shapes: rectangular, circular, triangular, and square. The volume of each geometrical shape is listed in Table S1. It is clearly seen that the fabricated solid drugs have good resolution. In addition, Figure 2b shows the EDS images of the circular solid drugs. The EDS result shows that the structure contains carbon and oxygen atoms. In addition, in order to check the versatility of the proposed method, sorbitol diacrylate, doxorubicin, and the photoinitiator were mixed, and sorbitol-based solid drugs were fabricated through the application of UV light, as shown in Figure S4.

**Figure 3:**
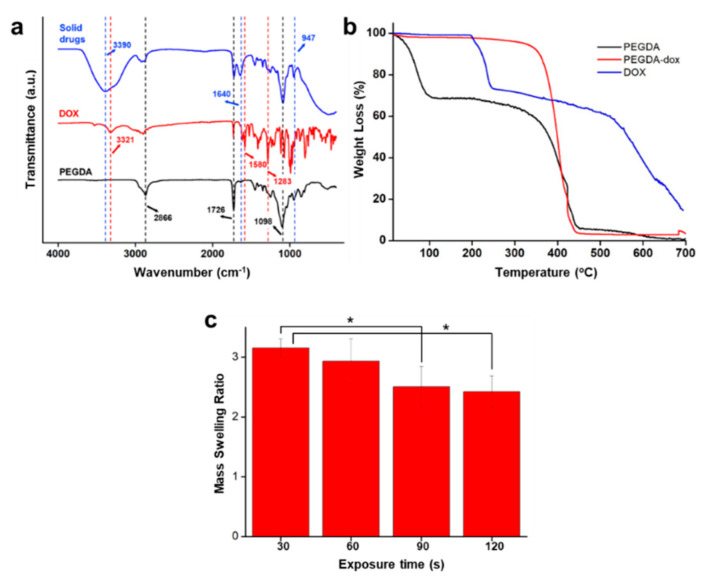
Characterization of solid drugs and their constituent materials: (**a**) FTIR; (**b**) TGA; (**c**) mass swelling ratio (* *p* < 0.05, Student’s *t*-test; n = 3).

FTIR was employed to evaluate the potential effects of mixing the drugs and hydrogel. Figure 3a shows the FTIR spectrum of PEGDA, doxorubicin, and solid drugs. The spectrum of PEGDA shows the C–H stretching vibration band at 2866 cm^−1^, C=O stretching vibration band at 1726 cm^−1^, and C–O stretching vibration band at 1098 cm^−1^ [30]. The absorption band of doxorubicin shows the broad band of O–H stretching at 3321 cm^−1^, C–C stretching at 1580 cm^−1^, and C–O stretching band at 1283 cm^−1^ [31]. In the solid drug spectrum, the broad peak of O-H stretching, C=O stretching, C=C stretching, C–O stretching, and C=C bending band are observed at 3390 cm^−1^, 1726 cm^−1^, 1640 cm^−1^, 1098 cm^−1^, and 947 cm^−1^, respectively. The spectrum of solid drugs indicates that it consists of the combination of the spectra of PEGDA and doxorubicin.

To further evaluate the thermal stability of the solid drugs and their constituent materials, analysis was performed by using TGA. As shown in Figure 3b, the initial weight loss was observed in PEGDA after 100 °C due to loss of water from the hydrophilic polymer chain [32,33], and further weight loss was observed after 350 °C. For the doxorubicin sample, the initial weight loss was observed after the temperature reached 200 °C, owing to the melting point of doxorubicin at 195 °C [34,35]. Further weight loss appeared after that. For the solid drug sample, the thermal degradation started at 400 °C; the mixing of the PEGDA and doxorubicin and their UV patterning strengthened the structure of the solid drugs, which increased their melting point. Thus, the sample had relatively better thermal stability.

The swelling ratio of the samples was observed, and the results are shown in Figure 3c. The swelling ratios for the solid drug under UV exposure times of 30 s, 60 s, 90 s, and 120 s were 3.16 ± 0.15, 2.93 ± 0.38, 2.51 ± 0.34, and 2.42 ± 0.27, respectively. Generally, the swelling ratio decreases as the UV exposure time increases [36]. Thus, the same results were found in this study. Herein, an increase in the UV exposure time resulted in a decrease in the swelling ratio. The decreasing value of the swelling ratio may be due to prolonged time leading to hardening of the samples, decreasing the ability to absorb the water. In addition, the digital images of dried and swollen samples are shown in Figure S5.

### 3.2. Drug Concentration Measurements

**Figure 4:**
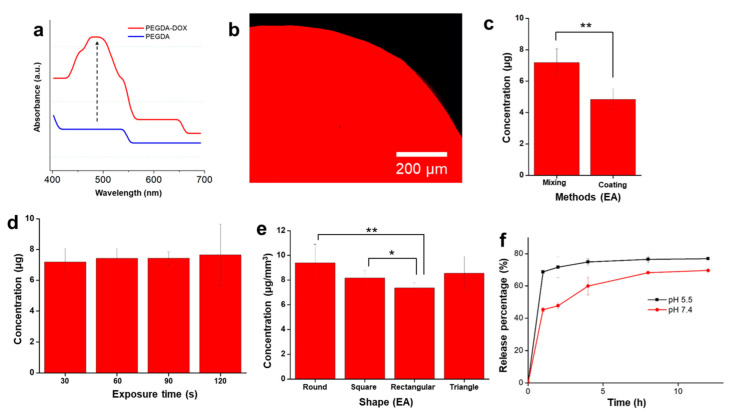
(**a**) Absorbance measurements for doxorubicin only and as solid drugs; (**b**) confocal image that shows the doxorubicin under fluorescence; (**c**) comparison of the concentration of the mixing and coating method (** *p* < 0.01, Student’s *t*-test; n = 3); (**d**) effect of exposure time on drug concentration; (**e**) the effect of shape on drug concentration (** *p* < 0.01 and * *p* < 0.05, Student’s *t*-test; n = 3); (**f**) drug release profile at two different pH values.

Absorbance was taken to confirm that doxorubicin was embedded in the PEGDA structure. The result is shown in Figure 4a. The PEGDA sample exhibited a low absorbance, whereas the PEGDA solid drug exhibited a doxorubicin peak absorbance that appeared at 480 nm. In addition, the confocal images of the patterned solid drugs were also taken wherein the red color shows the florescence intensity of the drug as shown in Figure 4b. The result indicated that the doxorubicin was indeed embedded properly in the solid structure.

The drug concentration in various methods, exposure durations, and polymer shapes was measured and evaluated. First, the drug concentrations obtained with the coating and mixing methods were compared. Polydopamine (PDA) has been used in many surface modifications [37]. Therefore, in this comparative study, the sample was coated with PDA for 6 h, and further coated with doxorubicin (15 mg/mL) for another 6 h. The result shown in Figure 4c demonstrates that the mixing method is likely to yield a higher drug concentration compared to the coating method. Moreover, the mixing method is preferable for preparation, being simpler and faster than the coating method. In addition, the exposure time was varied to evaluate the optimum exposure time for the fabrication of the solid drugs, as shown in Figure 4d. The exposure times to pattern the solid drugs ranged from 30–120 s. Although the concentration increased with an increase in the exposure time, the results show that the final concentration of the drugs was similar in all samples exposed to varying durations of UV light and no significant difference was found between the samples. Therefore, 30 s was chosen for further experiments. In addition, this study shows the versatility in patterning the drug in various shapes. The solid drugs were patterned into three other additional shapes: square, rectangular, and triangular. As shown in Figure 4e, the final drug concentration is dependent on the geometrical volume of the solid drugs. Based on our results, the measured concentrations are 9.38 µg/mm^3^, 8.16 µg/mm^3^, 7.35 µg/mm^3^, and 8.5 µg/mm^3^ for circular, square, rectangular, and triangular polymer shapes, respectively. In addition, the result of drug loading capacity is shown in Figure S6. The value of drug loading capacity as a function of UV exposure time of 30 s, 60 s, 90 s, and 120 s was 6.19 ± 0.76 µg mg^−1^, 6.73 ± 0.59 µg mg^−1^, 6.79 ± 0.32 µg mg^−1^, and 7.08 ± 1.84 µg mg^−1^, respectively. As the exposure time increases, the loading capacity increases. It seems that a long exposure time entrapped the drug, leading to a higher capacity than a short time. However, there are no statistical differences between all the samples.

In addition, the drug release experiments were conducted at two different pH conditions—5.5 and 7.4—resembling the difference pH for the gastrointestinal (GI) tract organs, whereas the pH of upper GI tract in maintained at ~pH 7 and lower GI tract (duodenum) around pH 5–6 [38]. The drug release was observed up until 12 h. The drug release profiles are shown in Figure 4f. Here, the drug release was conducted for circular samples only. Based on our results, the drug release was faster at lower pH than at higher pH. Up until 12 h, 76.88 ± 0.07% of the drug was released from the structure at pH 5.5, whereas at pH 7.4, 69.6 ± 1.34% of the drugs was released. The phenomena that happened may be interpreted that the lower pH value might cause faster degradation and the intensive structure destruction of the hydrogel, leading to faster drug release. In addition, the protonation of DOX may have occurred at an acidic pH, which increases its hydrophilicity and, thus, permeability across the hydrogels [39]. These results are in good agreement with the other reported studies on the drug release of hydrogel materials [40,41].

### 3.3. In Vitro Cell Studies

**Figure 5:**
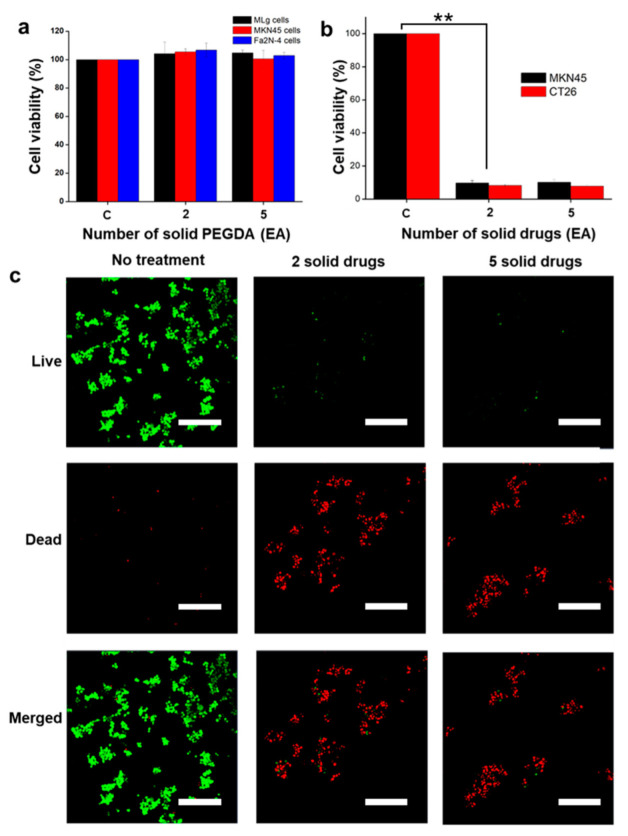
Cell studies assessing treatment with the fabricated PEGDA alone or with solid drugs: (**a**) Biocompatibility of PEGDA, when the treatment was conducted for 24 h against MLg cells, MKN45 cells and Fa2N-4 cells. The lack of reduction in cell viability indicates that the PEGDA is biocompatible; (**b**) Cytotoxicity of the solid drugs against CT26 and MKN45 cancer cells after 24 h. The reduction in cell viability indicates that the solid drugs can effectively release the drugs and successfully kill the cancer cells. (** *p* < 0.01, Student’s *t*-test; n = 3); (**c**) Live (green) and dead (red) cell imaging of the solid drugs against MKN45 cancer cells (scale bar: 200 µm).

The in vitro cell studies were performed by treating the PEGDA only to check its biocompatibility with solid drugs to evaluate the effectiveness of the drug release and therapeutic efficacy. The biocompatibility of patterned PEGDA is shown in Figure 5a; it was treated with MLg cells, MKN45 cells and Fa2N-4 cells by increasing numbers of PEGDA polymers (two, and five), with “C” depicting the control data. The result of the biocompatibility test shows no cell death, which indicates that the patterned PEGDA causes no harm to the cells and is compatible with living cells [42]. In addition, no significant differences was found. Figure 5b depicts the results of the treatment of cancer cells with solid drugs. Here, the cytotoxicity test was performed for 24 h by using two different cell types: MKN45 (stomach cancer cells) and CT26 (colon cancer cells). After treatment for 24 h, with two and five solid drugs, the cell viability was reduced significantly for both types of cells. Notably, the results indicate that the drug contained in the structures was successfully released and could effectively kill the cancer cells. Finally, the live and dead cell imaging was conducted (with MKN45 cells against solid drugs); the results are shown in Figure 5c. The green color represents live cells and the red color represents dead cells. It can be seen clearly that the cells are dead after treatment with the solid drugs. This proves that the solid drugs are extremely toxic to the cancer cells and demonstrate an excellent therapeutic effect.

## 4. Conclusions

In this study, the fabrication of solid drugs using polymeric hydrogel (PEGDA) and anti-cancer drugs (doxorubicin) was demonstrated through UV patterning. A wavelength of 365 nm was applied for 30 s to polymerize diacrylate oligomer. The solid drugs were successfully patterned into several geometrical shapes and verified drug dissolution and therapeutic efficacy. The absorbance results demonstrated that the drugs were successfully embedded into the solid structure. In addition, the circular shape resulted in a larger final drug concentration value of 9.38 µg/mm^3^. Thus, the proposed system shows potential versatility for the fine-tuned fabrication of various materials.

The in vitro cell studies showed that the patterned PEGDA samples exhibited excellent biocompatibility; moreover, when drugs were embedded in the structure of the hydrogel, they showed an excellent therapeutic effect. This means that the drugs inside the structure could be released effectively to kill the cancer cells. However, further studies are required in in vivo environments. In addition, although the embedded drugs can be released from the hydrogel structure, studies need to be conducted on various other types of drugs to evaluate further applications in personalized medicine. Furthermore, it is important to evaluate the degradation of PEGDA so that it does not remain in the body after the drug release.

## Data Availability

The data presented in this study are available on request from the corresponding author.

## Supplementary Materials

The following supporting information can be downloaded at: https://www.mdpi.com/article/10.3390/polym14132714/s1, Figure S1: Optical system that was used in this study; Figure S2: Illustration of the 3D-printed microchamber; Figure S3: The optical microscope of (a) PEGDA (b) solid drugs; Figure S4: The optical microscope of the patterned solid drugs based on Sorbitol diacrylate; Figure S5: The digital images of the (a) dried sample and (b) swollen sample of the solid drugs with 30 s exposure time (scale bar: 2 mm); Figure S6: The drug loading capacity of the solid drugs; Table S1: Volume of the patterned geometry.
