# Supplementary material for: Shape-Tunable UV-Printed Solid Drugs for Personalized Medicine
Source: Polymers (Basel). 2022 Jul 2;14(13):2714. doi: 10.3390/polym14132714 (PMC9269401; doi:10.3390/polym14132714)
Supplement: Supplementary file 1 [file polymers-14-02714-s001.zip › polymers-1762521-supplementary.pdf]

polymers

MDPI

Supplementary material

Shape tunable UV-printed solid drugs for personalized medicine

Bobby Aditya Darmawan[1,2], Sang Bong Lee[3], Minghui Nan[1,2], Van Du Nguyen[1,2], Jong-Oh Park[2,*] and Eunpyo Choi[1,2,*]

1 School of Mechanical Engineering, Chonnam National University, 77 Yongbong-ro, Buk-gu, Gwangju 61186, South Korea; bobbyaditdit@gmail.com (B.A.D.); myeonghea94@kimiro.re.kr (M.N.); nvdu81@gmail.com (V.D.N)

2 Korea Institute of Medical Microrobotics, 43-26, Cheomdangwagi-ro 208-beon-gil, Buk-gu, Gwangju 61011, South Korea;

3 THERABEST, Co., Ltd., Seocho-daero 40-gil, Seoul 06657, Korea; sblee@therabest.co.kr (S.B.L.)

* Correspondence: jop@kimiro.re.kr (J-OP); eunpyochoi@jnu.ac.kr (E.C).

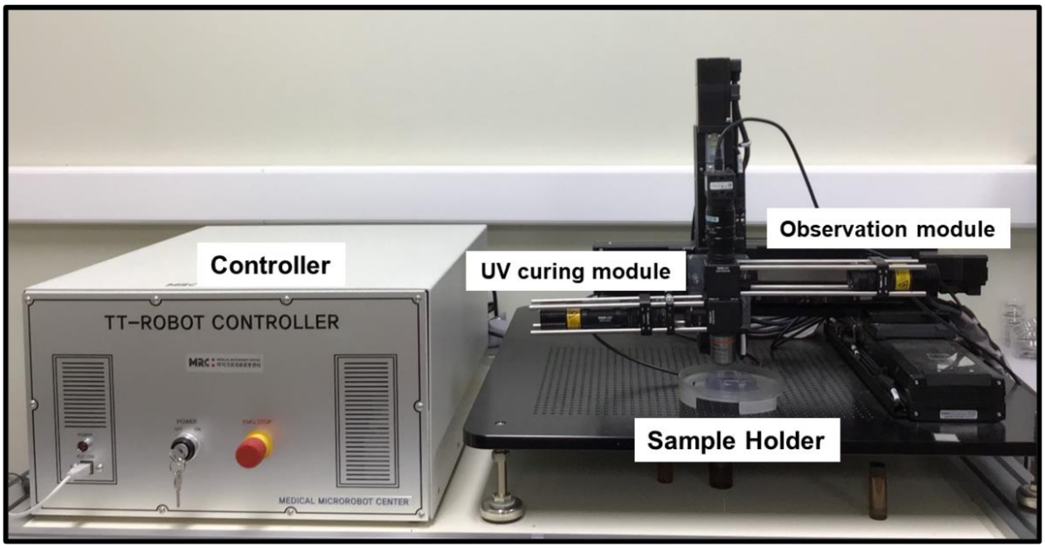

Figure S1. Optical system that was used in this study

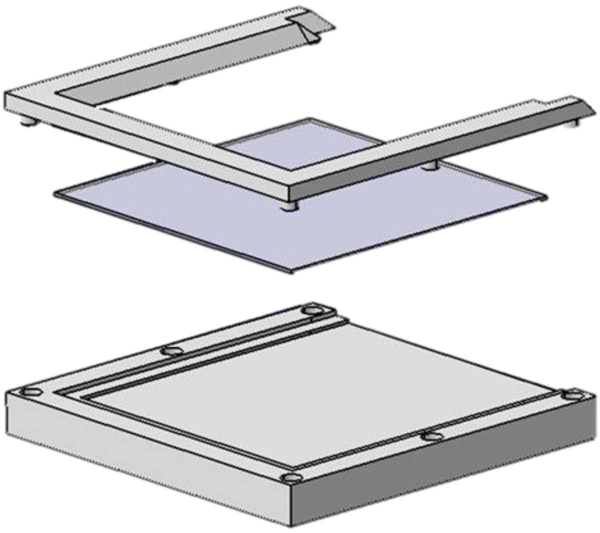

Figure S2. Illustration of the 3D-printed microchamber

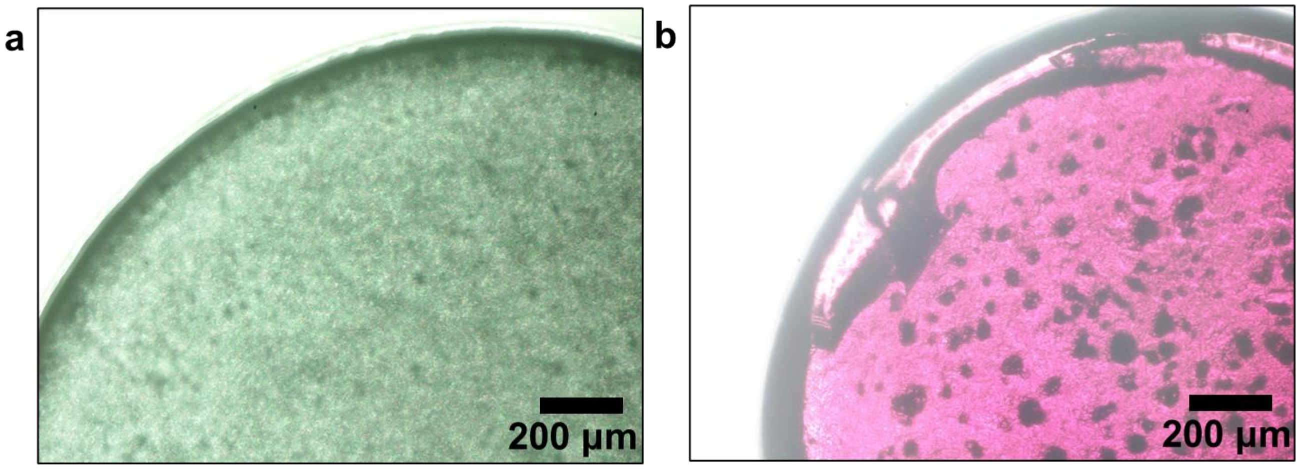

Figure S3. The optical microscope of (a) PEGDA (b) solid drugs

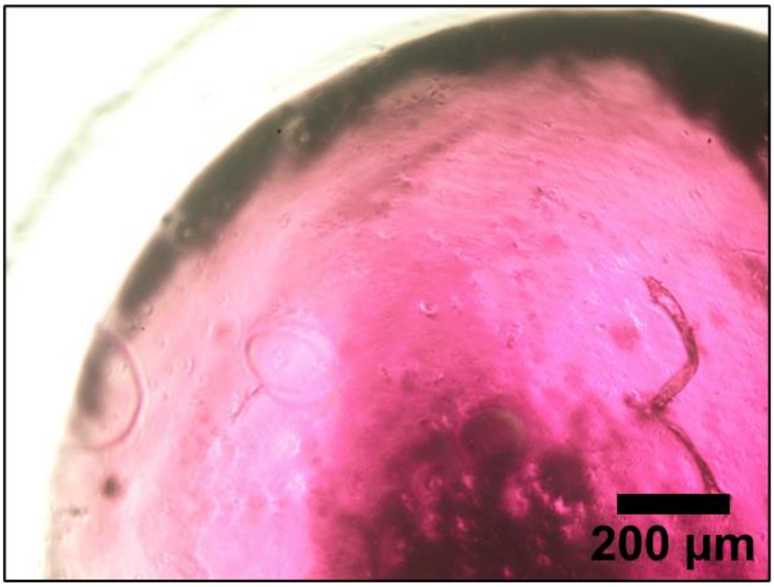

Figure S4. The optical microscope of the patterned solid drugs based on Sorbitol diacrylate

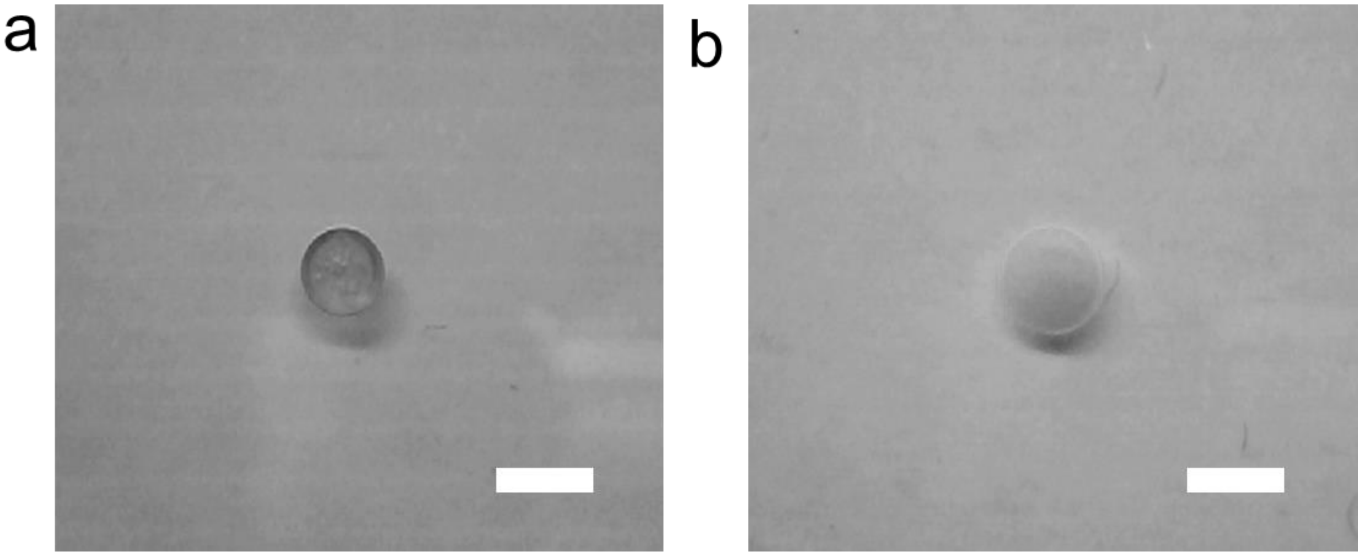

Figure S5. The digital images of the (a) dried sample and (b) swollen sample of the solid drugs with 30 s exposure time (scale bar: 2 mm).

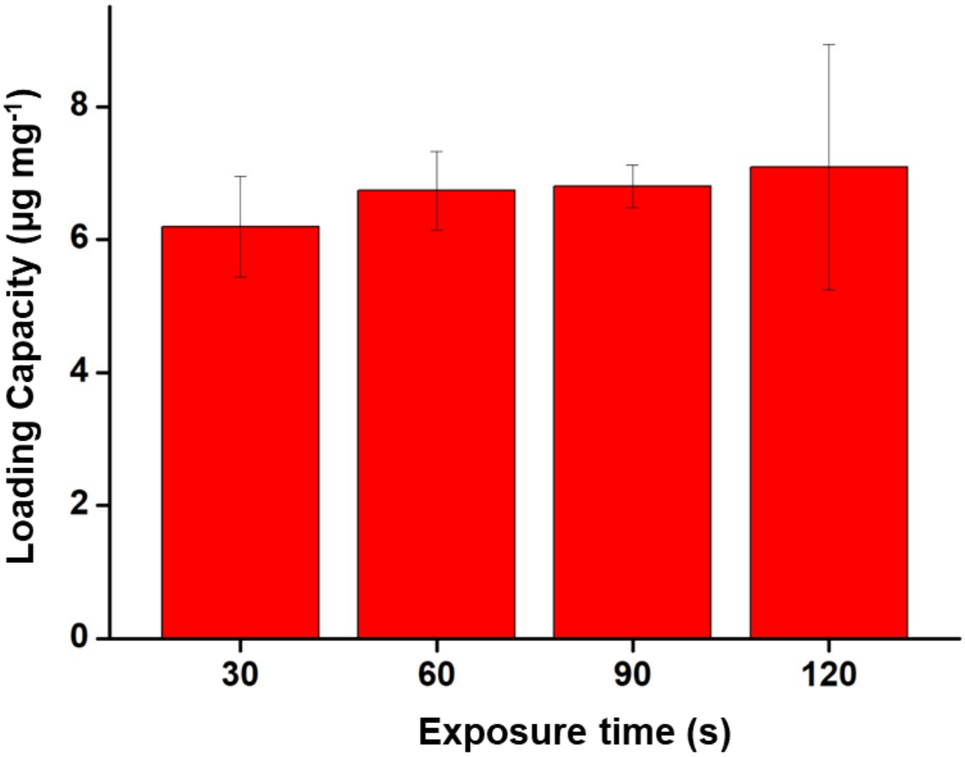

Figure S6. The drug loading capacity of the solid drugs.

Table S1. Volume of the patterned geometry

Shape	Volume (mm^3)
Round	0.739432
Square	0.81446
Rectangular	1.042903
Triangle	1.166614
